# Lost in Projection: Uncertainty is Misrepresented in Climate Risk and Vulnerability Assessments

**DOI:** 10.1111/risa.70331

**Published:** 2026-09-02

**Authors:** Patrick Curran, Kendrick Hardaway, Tom Logan

**Affiliations:** ^1^ Civil and Environmental Engineering University of Canterbury Christchurch New Zealand; ^2^ Biological and Agricultural Engineering University of Arkansas Fayetteville Arkansas USA; ^3^ Urban Intelligence Christchurch New Zealand

**Keywords:** adaptation, climate change, policy, risk, uncertainty

## Abstract

In practice, many climate change risk assessments fail to capture the true depth of uncertainty. Despite widespread scientific recognition of deep uncertainty (large ranges of possibility) in climate conditions, this nuance is often lost in translation to policy and planning contexts; instead, conditions are presented as single projections. This means that communities are making large‐scale infrastructure investments and long‐term policy commitments based on false precision, leaving them unprepared for climate surprises or potentially wasting resources and disrupting communities unnecessarily by overadapting. To examine how climate uncertainties are represented and accounted for in adaptation planning, we conducted a structured review of 39 climate risk and vulnerability assessments from across the world, using sea level rise as a case study. These documents inform policy that guides billions of dollars in infrastructure investments and shape community preparedness strategies. Our analysis reveals that only 54% of these documents correctly represent sea level rise as deeply uncertain. This issue is compounded when making decisions; 71% of decisions were made by misapplying scenarios as individual projections to plan for, rather than as a tool for exploring potential future conditions, directly contradicting their intended use. This demonstrates a gap between scientific understanding of climate uncertainty and planning practice. Addressing this gap requires improved uncertainty communication, moving beyond just quantifying uncertainty to also characterizing uncertainty. This must be done in conjunction with the support of decision makers to incorporate a stronger understanding of uncertainty into planning by using tools designed specifically for decision making in deeply uncertain environments.

## 1 Introduction

While research has examined sources of uncertainty in climate projections, comparatively little attention has been paid to how this uncertainty is represented and utilized in adaptation planning practice. Uncertainty is particularly pronounced in climate contexts, where the timing and scale of hazards and impacts remain uncertain (Schneider and Kuntz‐Duriseti 2002; Woodruff 2016). This uncertainty is compounded by wider factors, such as technological advancements, population dynamics, and economic shocks, making it impossible to know what future conditions will look like (Rauws 2017). If communities aim to be resilient, this uncertainty must be considered when planning for changing climate conditions (Hallegatte 2009). Yet the process by which these scientific characterizations translate into planning documents, and ultimately adaptation decisions, remain poorly understood, despite their potential significance for community resilience and resource allocation.

Historically, the response to planning for uncertainty has involved reducing uncertainty, aiming to establish a sense of certainty, often through projections or probabilistic models (Koshy et al. 2022; Mlakar 2009; Abbott 2005; Rauws 2017; Cox 2012). However, in climate contexts, certainty is unattainable, and what is often interpreted as certainty typically reflects simplified assumptions or constrained models rather than objective predictability (Petersen 2002; Rauws 2017). Designing a plan based on this “certainty” is called predict‐and‐act planning.

Plans designed using predict‐and‐act methods are inherently vulnerable to failure when future conditions diverge from expectations. For example, water supply modeling for Melbourne, Australia, suggested that the city had an adequate supply buffer through to 2020 under “severe climate change.” However, in the three years following the modeling, water supply failed to exceed half the long‐term average, and reached record lows in 2006 (Hulme et al. 2009). Another example can be found in 1990s, Dresden, Germany, where anticipated population decline led to the demolition of inner‐city housing, at the same time population numbers began to rebound (Wiechmann 2008). Similarly, the Channel Tunnel was planned based on a projection of 16.3 million passengers in its first full year of operation; actual ridership was only 7.1 million, a 56% shortfall (Chevroulet et al. 2007; Anguera 2006). This led to significant debt issues for operators, and a retrospective cost–benefit analysis indicated that the British economy would have been better off had the Tunnel never been constructed (Anguera 2006). Each of these plans failed because the future deviated from what they expected.

However, uncertainty is more than just the deviation from the expected value; this variation is just the manifestation of a far more complex and nuanced concept. Uncertainty encompasses a range of levels, from statistically quantifiable outcomes to complete ignorance (Courtney 2001; Marchau et al. 2019a). Some uncertainties are reducible through better data or modeling, while others are unknowable (Walker et al. 2003; Kiureghian and Ditlevsen 2009). Treating all uncertainty as if it were the same may result in the misapplication of planning tools and analytical methods. Recognizing and characterizing different types of uncertainty is therefore a critical first step in developing strategies that are appropriate for our decision‐making contexts (Kwakkel et al. 2010).

**FIGURE 1:**
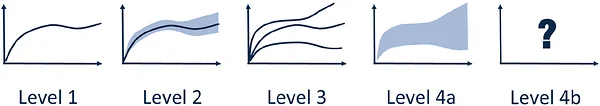
Levels of uncertainty (adapted Courtney (2003)).

Decision and risk sciences have developed frameworks for characterising uncertainty made up of several dimensions of uncertainty (Bevan 2022; Walker et al. 2003). One such dimension is the level of that uncertainty (Kwakkel et al. 2010; Walker 2011; Courtney et al. 1997). On a spectrum from determinism to total ignorance, uncertainty can be categorized into distinct levels that represent how precisely we can describe the future. The “Levels of Uncertainty” framework (Courtney 2001; Marchau et al. 2019a) splits this spectrum into five categories (1, 2, 3, 4a, and 4b), each requiring different analytical approaches and planning methods (Figure 1). Levels 1–4b are defined as

Level 1 uncertainties involve a clear and well‐defined understanding of the future, where uncertainties may exist but may not require precise quantification (Walker et al. 2013a; Marchau et al. 2019a). The future is often accurately described through a single deterministic model that yields a point value (Marchau et al. 2019a; Courtney 2003).
Level 2 uncertainties are represented statistically and involve situations where forecasts or trends have confidence intervals or multiple forecasts with associated probabilities (Walker et al. 2003, 2013a; Marchau et al. 2019a). This is a common representation of uncertainty and is frequently utilized in expected value and optimization problems (Marchau et al. 2019a).
Level 3 uncertainties are characterized by a set of plausible futures or scenarios, where assigning probabilities is not feasible, but it still may be possible to rank the scenarios (Walker et al. 2003; Marchau et al. 2019b). Such uncertainties typically arise when multiple plausible models exist or in situations involving unknown discrete outcomes, such as regulatory or judicial changes (Courtney 2003; Walker et al. 2013a).
Level 4a uncertainties can only be represented as a range of plausible outcomes (Walker et al. 2013a). This may occur when dealing with unstable macroeconomic conditions or global CO_2_ emissions (Courtney 2003).
Level 4b represents an unknowable future, where the mechanisms or functional relationships of the systems of interest are entirely unknown, leaving us with no means of defining a range of possible futures (Walker et al. 2003, 2013a; Courtney 2003).

Level 4a & 4b uncertainties are often described as “Deep Uncertainty.” Deep uncertainty is defined as:

“the condition in which analysts do not know or the parties to a decision cannot agree upon the appropriate models to describe interactions among a system's variables, the probability distributions to represent uncertainty about key parameters in the models, and/or how to value the desirability of alternative outcomes.” Lempert et al. (2003)

Climate change is a prime example of deep uncertainty (Sillmann et al. 2020; Stults and Larsen 2020; Bakker et al. 2017; Brock and Xepapadeas 2021; Wong and Keller 2017; Baum 2024; Styczynski et al. 2014; Marchau et al. 2019b; Hulme et al. 2009; Lempert et al. 2024). The range of uncertain conditions (including but not limited to carbon emissions, political and economic shocks, technological advancements, and population changes) that influence a complex social–ecological–technological system has created an environment that is unable to be described stochastically or by projections.

Given that climate impacts can only be described through a range of possibilities or an acceptance of not knowing, rather than a projection or stochastic output, it is impossible to use predict‐and‐act planning methods. Thus, under deep uncertainty, predict‐and‐act or optimization methods become obsolete (Lempert et al. 2003). Using these obsolete methods requires ignoring the uncertainty and may produce plans that may prove detrimental to communities (Christensen 1985; Zandvoort et al. 2018). Instead, deep uncertainty must be actively incorporated into climate adaptation planning methods (Zandvoort et al. 2018; Balducci et al. 2011). To incorporate deep uncertainty into decision making, plans must be designed to change with the uncertainties (e.g., adaptive planning as in Haasnoot et al. 2013) or be robust to the uncertainty (Lempert et al. 2003; Ben‐Haim 2019).

Currently, how decision makers understand and interpret uncertainty in climate risk assessments is not well understood. While there have been reviews into the source of uncertainty in climate risk assessments (see Stults and Larsen 2020; Ylhäisi et al. 2015; Jose and Dwarakish 2020; Kimei et al. 2024), these studies focus on where uncertainty originates rather than how “deep” it is. To date, there has been no structured review examining how the level of uncertainty is represented in climate risk and vulnerability assessments.

Sea‐Level Rise (SLR) provides an ideal case study to examine how uncertainty is represented in climate risk and vulnerability assessments because it exemplifies the deep uncertainty challenges facing climate adaptation. Possible future SLR spans wide ranges due to fundamental uncertainties in ice sheet dynamics, regional variations, and the emissions scenarios (Kopp et al. 2023; Hu and Bates 2018). Yet coastal communities must make immediate infrastructure and policy decisions with decades‐long consequences. The way uncertainty around SLR is represented and communicated in risk assessments directly influences whether communities can develop appropriate adaptive responses. Therefore, we ask:

 How is uncertainty around SLR represented in climate risk and vulnerability assessments?
 What are the implications of this representation for adaptation planning?

## 2 Method

To assess how uncertainty is represented in climate risk and vulnerability assessments, we conducted a structured document review of 39 climate change risk and vulnerability documents. Our focus was on how precisely SLR was represented as characterized by the levels of uncertainty framework (see page 3 for definitions). In this section, we first outline the document selection process and then outline how each of the representations of uncertainty was coded.

### 2.1 Document Selection

**FIGURE 2:**
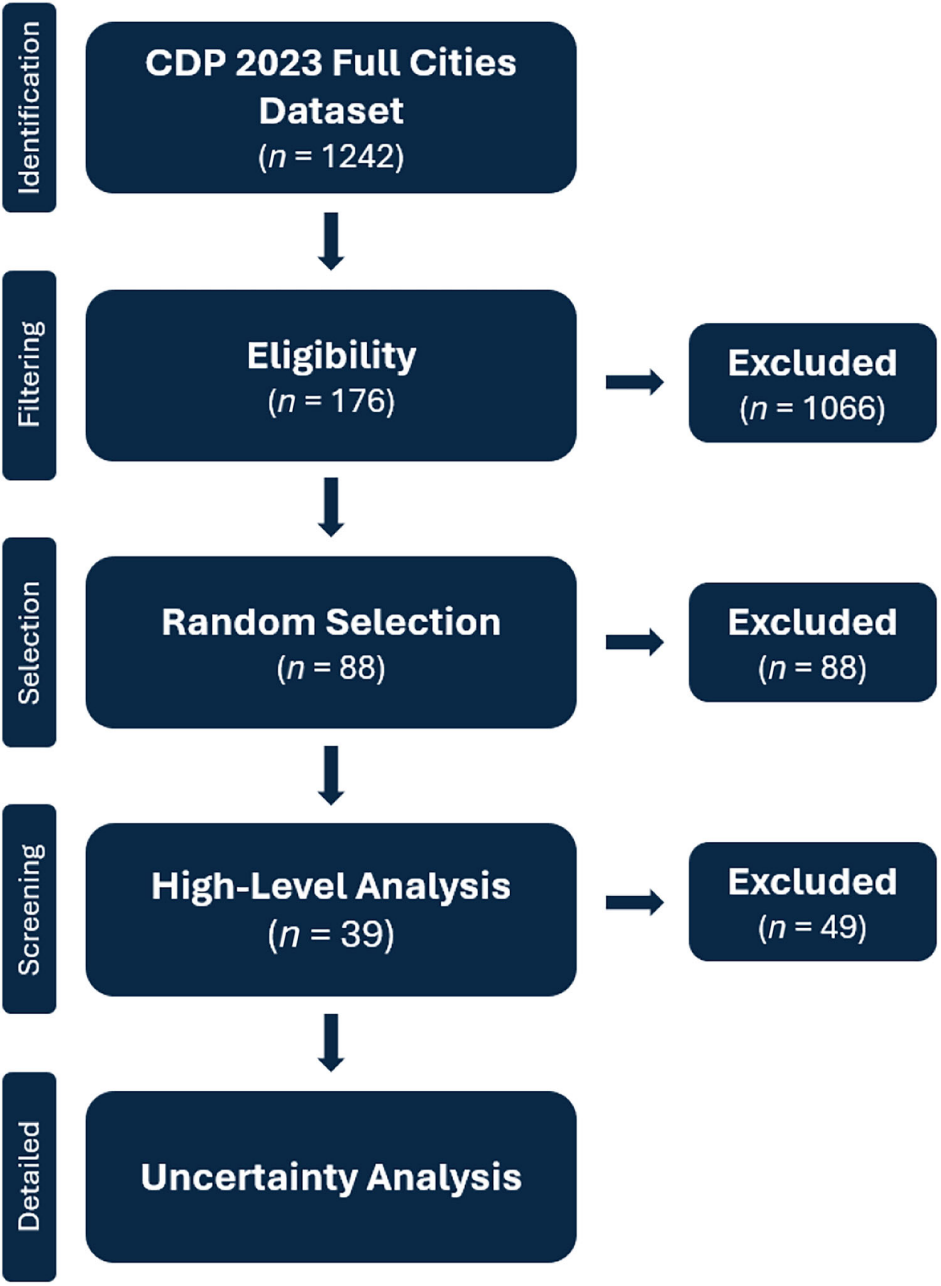
The structured review process (adapted from PRISMA (Page et al. 2021)) conducted to analyze how uncertainty is presented in climate change risk and adaptation documents.

The document selection process was adapted from PRISMA guidelines (Page et al. 2021) and comprised several stages, as illustrated in Figure 2.

#### 2.1.1 Document Identification

Documents were identified through the Carbon Disclosure Project (CDP), a global nonprofit that collates climate reporting documents from companies, capital markets, cities, states, and regions. We used the 2023 full cities database, which was the most current database available at the time of analysis and included 1242 documents published through 2022. The CDP database was selected because it has a database of climate risk and vulnerability assessments from diverse geographical contexts, providing insights into the global understanding of uncertainty.

#### 2.1.2 Document Filtering

Following document identification, a multistage filtering process was applied to refine the initial data set:

1. Topic filtering: Documents tagged as “Climate Risk and Vulnerability” were selected, as these were most likely to include representations of future conditions describing hazards that cities may encounter.
2. Temporal and accessibility filtering: Documents were further filtered to include only:
  - Recent publications (published after 2018)
  - Documents in English
  - Publicly available documents

This filtering process left 176 documents. While these criteria ensured comparability, currency, and accessibility, they also introduced limitations. Restricting the review to English‐language reports may exclude culturally or regionally specific approaches to conceptualizing and managing uncertainty. Similarly, focusing on a 2018–2022 publication window provides a snapshot of how uncertainty is handled but does not capture longer‐term trends. Limiting the data set to publicly available documents may also introduce a reporting bias, as some cities may produce more detailed or technically advanced materials that are not released publicly.

#### 2.1.3 High‐Level Analysis

Half of the remaining documents (n=88) were randomly selected for high‐level analysis. Documents were screened for inclusion of SLR content using the search terms “sea level rise,” “sea‐level rise,” and “SLR.” The documents that included these representations of SLR were then subjected to detailed analysis.

#### 2.1.4 Detailed Analysis

**FIGURE 3:**
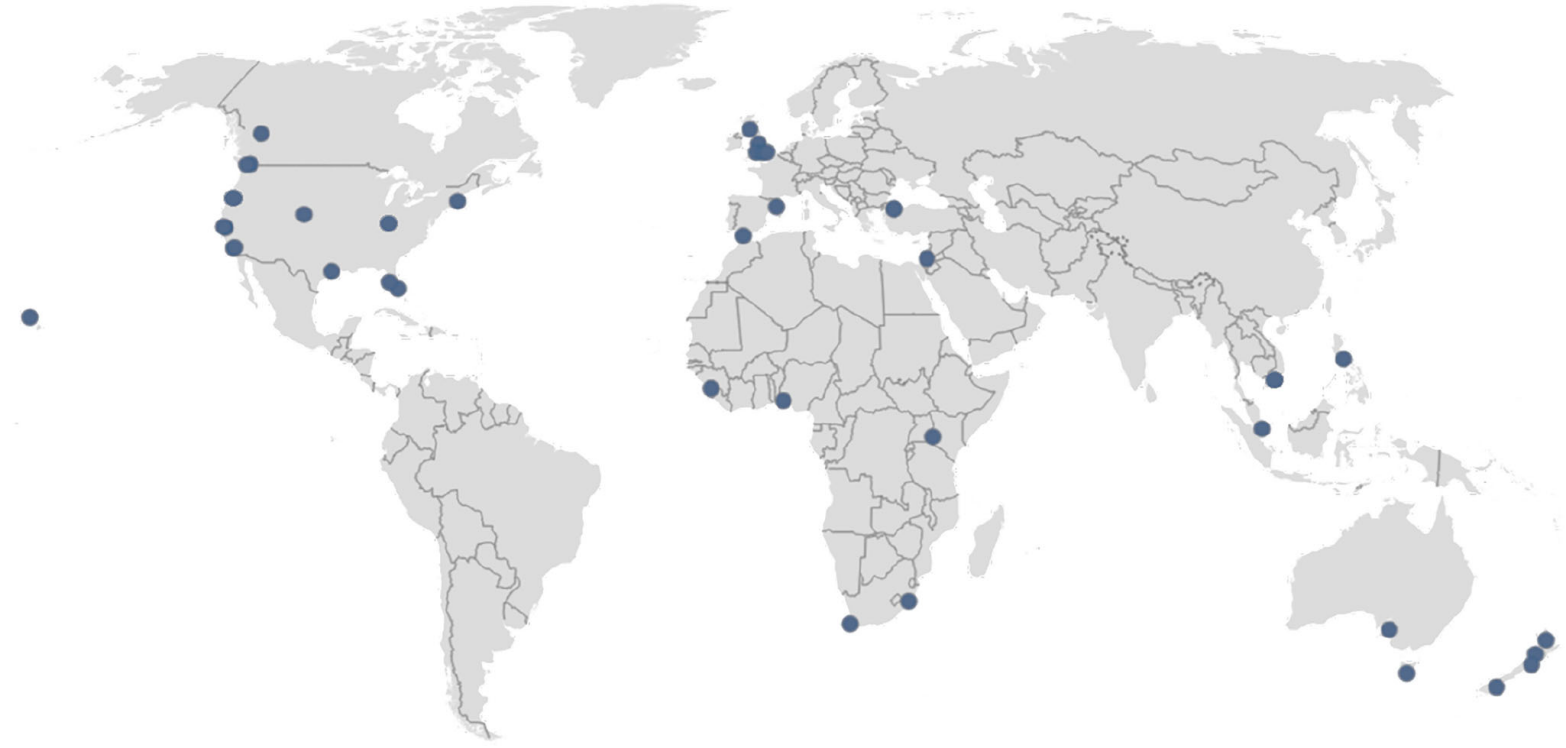
The geographical spread of documents included in the detailed analysis of uncertainty.

After screening 39 unique documents remain (see Appendix A for complete list). These documents were submitted by 37 separate cities, with Cape Town, South Africa and Saanich, Canada, both contributing two documents. The spatial distribution of the included documents can be seen in Figure 3. As expected, the final data set predominantly comprised coastal cities. Three documents from noncoastal cities (Boulder, Colorado; Bloomington, Indiana; and Kisumu County, Kenya) mentioned SLR. In the Boulder and Kisumu reports, SLR was referenced early as an example of climate impacts to establish context, demonstrating that SLR is a universally understood climate hazard, which supports our decision to focus specifically on SLR uncertainty. In Bloomington, a single SLR projection was used to show how the population may change as a result of climate migration. While Boulder, Bloomington, and Kisumu County may not integrate SLR into their decision making, they still provide a datapoint on how uncertainty is understood by and communicated to decision makers.

### 2.2 Uncertainty Coding

A detailed review of the uncertainty in each of these documents was conducted. A log of each instance of potential SLR in each document was created using the search terms “sea level rise,” “sea‐level rise,” or “SLR.” Each of these instances was classified according to a level of uncertainty:

Level 1 Single future presented, often as a “forecast.”
Level 2 Probabilistic language used (e.g., “likely,” “mean,” “average,” or percentage values).
Level 3 Multiple plausible future conditions presented using Representative Concentration Pathways (RCPs), Shared Socioeconomic Pathways (SSPs), or other scenario sets. Note: If scenario sets captured uncertainty across different hazard types (e.g., one SLR scenario, one storm scenario, one heatwave scenario) but not within‐hazard uncertainty (multiple SLR scenarios), it is classified as a Level 1 uncertainty.
Level 4a Ranges of possibilities without stochastic weighting or incremental SLR values without specified timeframes.
Level 4b specific acknowledgment of uncertainty and the inability to represent it.

Finally, each representation was tagged based on whether it was used in a decision‐making component or policy recommendation. Decision‐use representations occur when uncertainty is invoked to justify, frame, or guide a particular planning, policy, or engineering decision.

1. A policy, design, or planning action was proposed or justified in relation to that uncertainty;
2. The causal link between the uncertainty and the action was either direct (e.g., “based on projected sea level rise, we recommend…”) or implied through framing (e.g., “to account for future inundation, use the NOAA Intermediate High scenario…”).

To mitigate potential subjectivity in the uncertainty or decision‐use classifications, two reviewers coded the levels individually. Disagreements between reviewers were resolved through discussion. These discussions were around several edge cases; discussed in detail in Appendix B. Having established our analytical framework for classifying uncertainty representations, we now examine the patterns that emerged from our analysis of 39 climate risk and vulnerability assessments.

## 3 Results and Discussion

To understand how uncertainty around SLR is represented in climate risk and vulnerability assessments (RQ1), we first look at the range of distinct representations (repeated use of the same representation was counted once, whereas the use of multiple, distinct representations, such as two different sets of scenarios, was counted separately at the same uncertainty level) of uncertainty within each document across our 39‐document sample before looking at the distribution of levels of uncertainty across the documents. We then compare the distribution of how uncertainty is represented to the distribution of uncertainties tagged as decision‐use to understand the implications of these representations for adaptation planning (RQ2).

### 3.1 (In)Consistency of Representations Within Risk and Vulnerability Assessments

**FIGURE 4:**
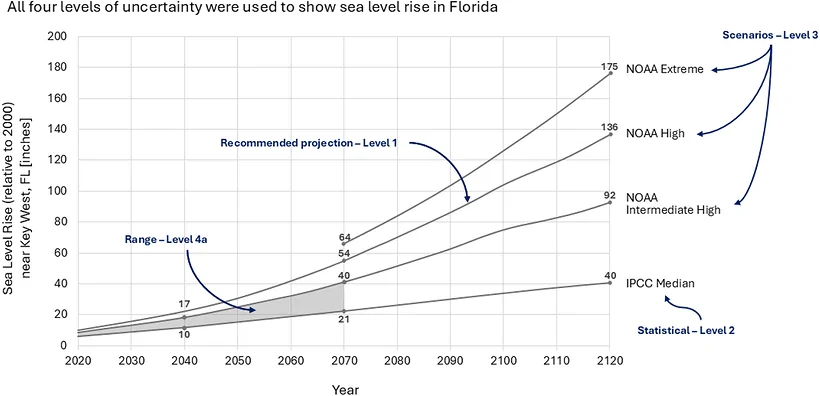
A stylized and annotated “Unified Sea Level Rise Projections” for Florida showing levels 4a, 3, 2, and 1 uncertainties in one figure (Southeast Florida Regional Climate Change Compact Sea Level Rise Work Group (Compact) 2020).

On average, each of the climate risk and vulnerability assessments had 3.97 distinct representations of uncertainty. While 10 of the documents (25.6%) had consistent uncertainty information throughout (one single representation); *South Palm Beach* and *Alameda* both had 11 distinct representations. This range of representations suggests there are significant inconsistencies in how uncertainty is represented within individual planning documents. For example, consider the *Unified Sea Level Rise Projection Southeast Florida* document, where four different levels of uncertainty were presented in one figure (Figure 4):

Level 1 Uncertainty. For long‐term planning “beyond 2070,” the *Unified Sea Level Rise Projection Southeast Florida* recommends using the NOAA High curve as a single projection for design purposes. This transforms a scenario (originally part of a set of six (Sweet et al. 2017)) into a deterministic projection to plan for.
Level 2 Uncertainty. The IPCC “median” projection was included and introduces probabilistic language, described as “the most likely average sea level before 2070.”
Level 3 Uncertainty. NOAA High, NOAA Intermediate High, and from 2070 onward, the NOAA Extreme scenario represent plausible futures based on varying assumptions about emissions and climate dynamics. In this case, only three of the six scenarios developed by NOAA were presented (Sweet et al. 2017).
Level 4a Uncertainty. For shorter time horizons, decision makers are directed to use the shaded range between the IPCC median curve and the NOAA Intermediate High curve. However, when making a decision, “the adaptability, interdependencies, and costs of the infrastructure should be weighed to *select a projection value* between the IPCC Median and the NOAA High curves,” and as such, the decision‐use component of this Level 4 representation was classified as a Level 1. Examples of how level 4 uncertainties could be included in decision making are presented in Section 3.3.3. For more solutions on dealing with Level 4a uncertainty, see Section 3.3.3.

Similar patterns of the inconsistent use of uncertainty were observed across multiple documents. For instance, the *Glasgow Climate Adaptation plan* used UKCP18 scenarios (Level 3) to explore possible SLR but then, under the guise of the precautionary principal (discuses in Section 3.3), wrote the adaptation plan based on only the high emissions scenario (Level 1), then used an SLR likelihood score (Level 2) when comparing different hazards.

Another example of the range of distinct representations of uncertainty in a document is the *Alameda Climate Action and Resiliency Plan*. Within this document, SLR was presented as:

- RCP 8.5 with probability bounds (Level 2).
- “24 inches of sea level rise will occur in 2050” (Level 1).
- “12 inches of sea level rise between 2030 and 2040” (Level 4a).
- Economic analysis was conducted using a different scenario (Level 1).
- Infrastructure modeling recommendations mentioned using future SLR scenarios (Level 3), but provided few details on the scenarios.
- Guidance suggested giving “more weighting to higher GHG emissions scenarios” (Level 2).

These examples illustrate how uncertainty can become fragmented and inconsistent across the risk and vulnerability analysis documents. This creates potential confusion for both planners and decision makers about the level of uncertainty underlying their decisions. From the literature, several factors may contribute to the inconsistent representation of uncertainty within individual documents including: the timescale, the information source, the source of uncertainty, political or strategic framing, and the misapplication of climate scenarios, though this list is not exhaustive.

The following subsections explore these factors in more detail with examples from the documents investigated. No examples of political or strategic framing were identified in the analyzed documents. However, this does not necessarily indicate its absence, as such framing operates through the presentation and communication of information and is therefore difficult to distinguish from other forms of misunderstanding. Strategic messaging can influence the level of uncertainty conveyed, even when the underlying evidence remains unchanged (Kuklicke and Demeritt 2016). Documents may emphasize urgency by using high‐end scenarios with deterministic framing (Level 1) to make decision makers appear more confident. Alternatively, decision makers may later choose to play up the uncertainty to provide a scapegoat when a plan fails (Kuklicke and Demeritt 2016; Allan and Curtis 2005).

#### 3.1.1 Timescale

Uncertainties compound over time, creating a future that is more uncertain the further out you try to predict (Hawkins and Sutton 2009). For example, Kadikoy Municipality suggested a wide range of sea‐level rise between 45 and 75 cm by 2100. In the short term, however, point projections of sea level rise may still be appropriate. This is due to the lag between greenhouse gas emissions and their full impact on the climate system, meaning that short‐term sea level rise is largely “locked in” based on current conditions (IPCC 2023a). As a result, there is significantly less uncertainty in projections for the near future. Consequently, near‐term decisions may justifiably rely on single projections or narrow uncertainty ranges (Levels 1–2), whereas longer‐term planning requires a broader range to capture the uncertainty (Level 4a).

Interestingly, the opposite of this is true in several of the assessments. For example, the *Unified Sea Level Rise Projection: Southeast Florida* recommends planning for a range of possibilities (Level 4a) up until 2070, and from 2070 onward recommends planning to a single projection (Level 1). Similarly, the *Alameda Climate Action and Resiliency Plan* presents an inconsistent view of uncertainty, projecting 24 inches of sea level rise by 2050 (Level 1), yet only 12 inches sometime between 2030 and 2040 (Level 4a).

#### 3.1.2 Information Source

Documents frequently draw from multiple data sources. For example, *Unified Sea Level Rise Projection: Southeast Florida* refers to both the IPCC and NOAA scenarios. Each data source employs different modeling assumptions and approaches to dealing with uncertainty. The IPCC data provided a singular median projection, whereas the NOAA data provide at least three scenarios. In the case of Alameda, California, they aligned their projections with state‐level data on sea‐level rise guidance, directly influencing how the city treated uncertainty. Auckland, meanwhile, performed their own study on sea‐level rise to create a range of likely sea‐level rise scenarios, then incorporated RCPs and a general statement on accepting uncertainty beyond the scenarios generated. This leads to conflicting uncertainty language and levels across the document, with different assumptions about emissions trajectories, ice‐sheet dynamics, or storm modeling being presented simultaneously.

#### 3.1.3 Source of Uncertainty

The source (where the uncertainty manifests within the system (Walker et al. 2003; Kwakkel et al. 2010)) of the uncertainty also changes how uncertainty is represented. For example, *Climate change risks for London* presented two scenarios: low emissions and current emission levels (Level 3); and then gave a very likely range (Level 2) around each of them. These different levels were used to capture different sources of uncertainty. The two scenarios capture the context uncertainty. Context uncertainty is everything outside of the system model and may include the economic, environmental, political, social, and technological changes that provide the underlying context to the climate models (Walker et al. 2003). The very likely range around these scenarios captures the internal variability and model uncertainty within the scenarios (IPCC 2023a). The Coupled Model Intercomparison Project (CMIP) scenarios, adopted by the IPCC (henceforth called the IPCC scenarios), are developed through ensemble modeling of several Global Climate Models (GCMs). Monte Carlo simulations create a distribution that captures the internal variability of the climate system. The distributions for each model are combined to capture the model structure uncertainty (IPCC 2023a).

Classifying different sources of uncertainty offers a granular understanding of the overall uncertainty. Taking this more granular approach to understanding, the uncertainty provides insights into how to address the individual components contributing to it. The overall uncertainty of a system will match or exceed the highest uncertainty level of its components.

#### 3.1.4 Misapplication of Climate Scenarios

The misapplication of climate scenarios can also lead to inconsistent representations of uncertainty. Scenarios (Level 3) are commonly used to communicate climate uncertainty by both the IPCC (2021) and NOAA (Sweet et al. 2017), and provide a useful way to explore uncertainty and understand the potential impacts of climate change (Moss et al. 2010). However, the uncertainty they represent can be lost when scenarios are misapplied. Two forms of scenario misapplication were identified: a single scenario can be selected (Level 1) (elaborated on in Section 3.3), or probabilistic methods can be applied to the scenarios (Level 2).

A probabilistic method being applied to the IPCC scenarios can be seen in Figure 4 from the *Unified Sea Level Rise Projection: Southeast Florida* where they take the median IPCC scenario. Originally, the Special Report on Emissions Scenarios (SRES), published in 2000 (Nakicenovic and Swart 2000), presented four primary scenarios, with no central case, specifically to reinforce the idea that scenarios should be considered plausible alternative futures while not presenting a central storyline that could be perceived as the most likely. Taking this approach aligns with the broader scenarios literature (Goodspeed 2020; Meristö 2017). However, Assessment Report 6 (AR6) had five SSPs introducing a central scenario (IPCC 2023b). This change in approach by the IPCC then opened the door to this type of misapplication.

Each of these inconsistencies found within individual documents reveals important challenges in uncertainty communication. Taken together, they highlight how fragmented approaches at the document level can influence, and at times obscure, the uncertainty in climate science. We now examine the overall distribution of the level of uncertainty across our sample to provide insights into broader patterns in planning practice.

### 3.2 Representations of Uncertainty Across Risk and Vulnerability Assessments

To minimize the noise introduced by the inconsistencies within documents, we characterized each document by its highest level of uncertainty representation. This approach provided the most optimistic assessment of how well planning documents align with scientific understanding of deep uncertainty, as it credits documents for their representation that most closely aligns with deep uncertainty rather than their simplified applications. By adopting this method, we avoid, as much as possible, the downplaying of the level of uncertainty through strategic framing or at short timescales, and look at the uncertainty of SLR as a whole (not as individual sources). However, this approach may obscure certain misapplications of scenarios and potentially understate the prevalence of lower levels of uncertainty.

**FIGURE 5:**
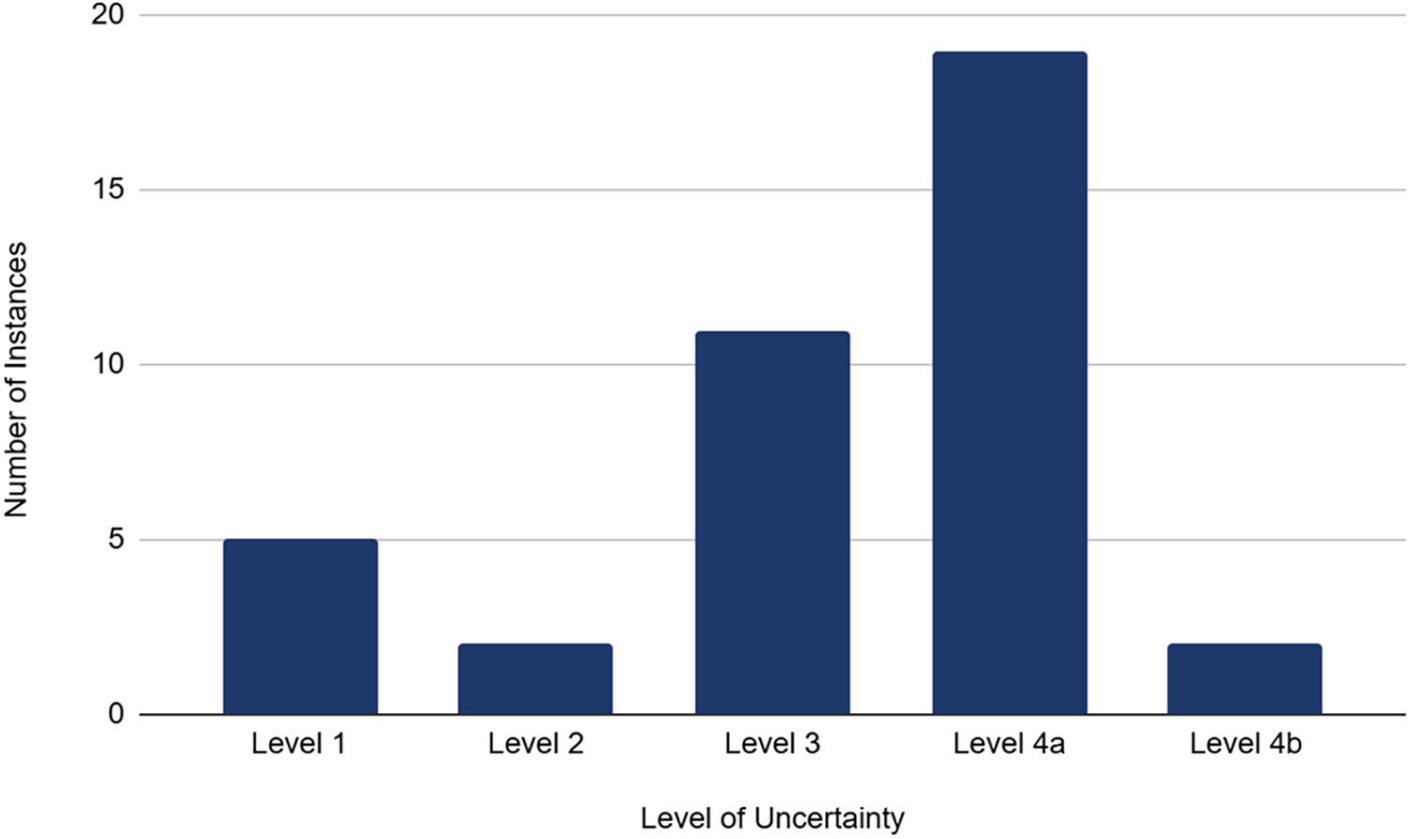
A bar chart showing the instances of representations of future conditions broken down by each level of uncertainty.

Distribution of uncertainty representations Figure 5 shows the distribution of uncertainty levels in SLR. There is a clear peak at Level 4a, appearing in 19 documents (48.7% of the sample). When combined with Level 4b representations (two documents, 5.1%), a total of 21 documents (53.8% of the sample) represent SLR as deeply uncertain. This suggests that only half of climate risk and vulnerability assessments acknowledge—at their highest level of analysis—that SLR represents a deeply uncertain phenomenon.

Level 3 uncertainties, which involve scenarios, are the second most common representation, appearing as the highest level in 11 documents (28.2%). Level 1 deterministic representations are the highest in five documents (12.8%), while Level 2 probabilistic approaches are found in only two documents (5.1%).

While only 28.2% of the documents classify Level 3 uncertainties as the highest level of uncertainty, the overall use of scenarios is more widespread. Nineteen documents classified as Level 4a or 4b also include some form of scenario. This prevalence is not surprising, as the IPCC produces climate change data through scenarios like RCPs and, more recently, SSPs (Pörtner et al. 2023). Several documents also used UKCP18 projections (which are based on RCPs), NOAA scenarios, or a mix of RCPs and NOAA scenarios.

Some of these level 4 documents employed scenarios but included explicit acknowledgments of their limitations. For instance, the *Preliminary Strategic Climate Risk Assessment for British Columbia* (Ministry of Environment and Climate Change Strategy 2019) noted: “The scenarios do not necessarily represent the most severe version of each risk event that could occur today or be possible by 2050; more severe and less severe versions of each scenario are also possible.” Similarly, *Climate change risks in Auckland* (ARUP 2019) highlighted: “The current projections may accelerate at an unknown rate, so that effects projected for the next century ‐ such as sea level rise ‐ may take place over a much shorter time frame.” Such acknowledgments reflect an understanding of scenario limitations, even when scenarios remain the primary analytical tool.

The relative scarcity of Level 2 (probabilistic) representations may be partly explained by the prevalence of scenarios in climate science. Scenarios in their original form contain no inherent probabilistic information; they represent plausible storylines rather than likelihood‐weighted predictions. Instead, decision makers either use scenarios with appropriate caveats (Level 4) or select single scenarios for practical application (Level 1), as discussed in Section 3.3.

While the prevalence of scenarios is understandable, given their use by the IPCC and other bodies to communicate climate information, this does not change the fact that SLR is deeply uncertain. Instead, the scenarios are designed as a tool to explore the level 4 uncertainties rather than as a description of the possible futures. This nuance appears to be lost in nearly half of climate risk and vulnerability assessments. If this disconnect between how uncertain SLR is in reality versus how they are presented continues into the decisions being made, there could be serious impacts for communities, as plans will not be robust (Walker et al. 2013b).

### 3.3 Uncertainties Used in Decision Making

**FIGURE 6:**
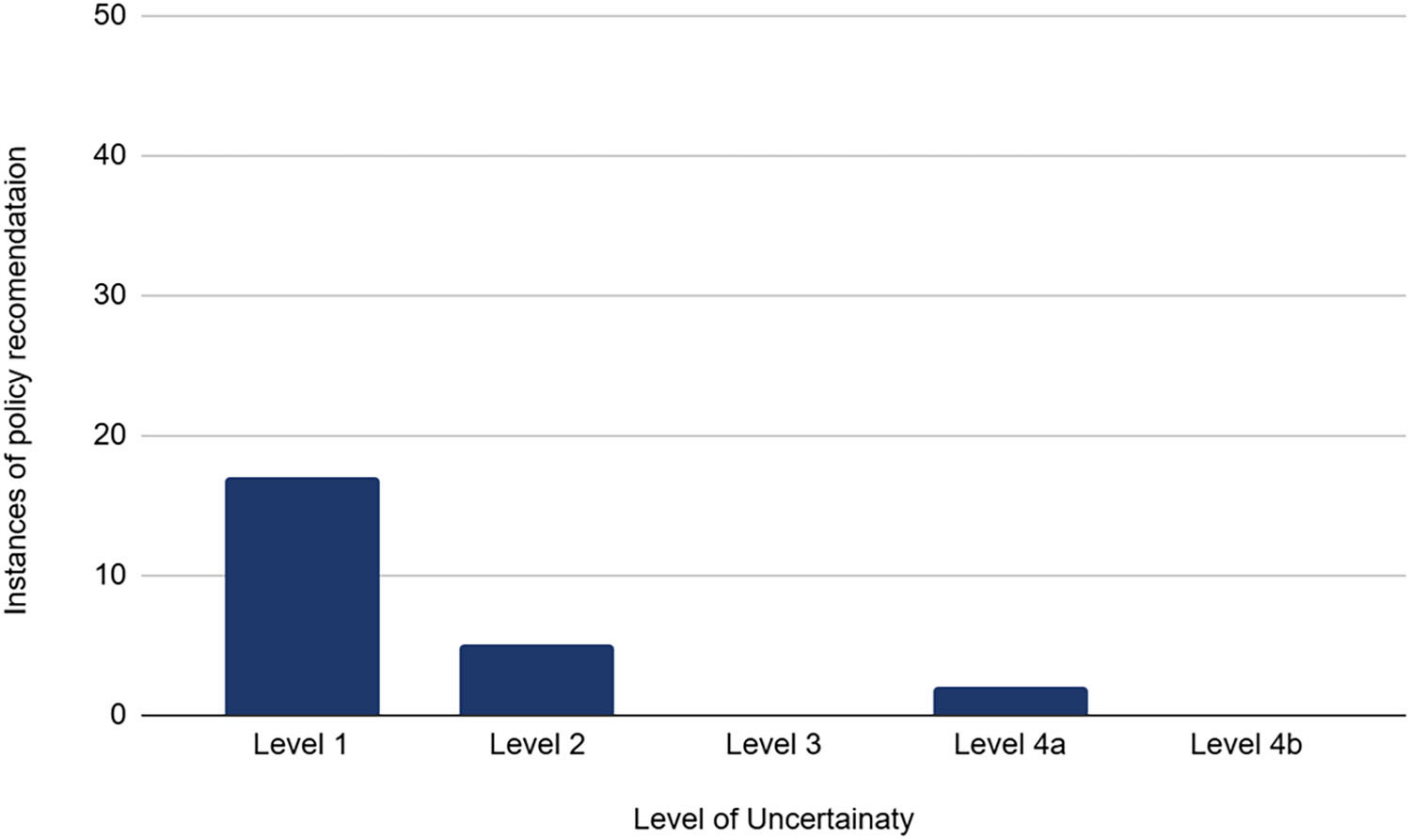
A bar chart showing the instances of policy recommendations broken down by each level of uncertainty.

Having established that deep uncertainty in SLR was not adequately recognized by practitioners, the next step was to examine how this understanding (or lack thereof) influences decision making. To do this, we analyzed the representations of uncertainty that were explicitly used to inform policy recommendations or guide climate adaptation decisions (n=24), as shown in Figure 6. These cases illustrate how uncertainty is operationalized in practice and reveal a significant shift in its representation when concrete action is required.

A comparison of Figures 6 and 5 highlights three notable patterns. First, Level 3 (scenario‐based) uncertainties are absent. Second, Level 1 (deterministic) approaches become the dominant representation of uncertainty. Finally, there is a reduction in the proportion of uncertainties at Level 4 (deep uncertainty). These patterns are explored in more depth in the following subsections.

#### 3.3.1 Absence of Level 3

Level 3 (scenario‐based) representations disappear entirely when decisions are made. While 28.2% of documents in the broader sample used scenarios as their highest form of uncertainty representation, none of the 24 decision‐focused cases relied on Level 3 approaches. This suggests a gap in the practical application of scenario‐based methods, with decision makers either unsure of how to incorporate multiple scenarios or finding them too complex for policy contexts. Instead, single scenarios are often selected and treated deterministically, which explains the sharp increase in the use of Level 1 uncertainties.

To make decisions under a level 3 uncertainty, a scenario‐based approach should be used (Walker 2011). It is often recommended that three to seven scenarios should be used to balance capturing possible futures with what can practically be used in decision making (Xiang and Clarke 2003). An even number of scenarios is also recommended to avoid presenting a central scenario (Goodspeed 2020; Nakicenovic and Swart 2000; Meristö 2017).

Scenarios can be developed in three ways: normative, predictive, and exploratory (Börjeson et al. 2006). Predictive scenarios are based on the question “what will happen?” and attempt to predict what will happen if some action is taken (Maier et al. 2016). Normative scenarios ask “how can a specific target be met?” in an attempt to identify the steps to take to get to a preferred future (Maier et al. 2016). Exploratory scenarios are used to understand what could happen using “what if?” questions (Maier et al. 2016). Predictive and Normative scenarios are becoming less common as they can imply a degree of control over future conditions (Goodspeed 2020). In contrast, exploratory scenarios are designed specifically to capture uncertainty in future conditions and, as such, are also useful when faced with Level 4 uncertainties.

The IPCC scenarios (RCPs and SSPs) take an exploratory approach and are a tool to explore deep uncertainty rather than an attempt to present all possible futures. Exploratory scenarios must be examined collectively rather than treated as projections of the future. Using scenarios in this manner matches more closely with the concept of scenario planning (Goodspeed 2020; Avin and Goodspeed 2020; Peterson et al. 2003). However, in practice, the IPCC scenarios are often cherry‐picked and planned against shifting the decision‐making process to level 1 uncertainty.

#### 3.3.2 Shift Toward Level 1

Seventeen (71%) of the decisions were made using Level 1 uncertainty. These 17 decisions were made across 10 documents, with 60% of the documents selecting a future from a set of scenarios. The *Otago Climate Change Risk Assessment*, *Climate Ready O'ahu*, and the *Alameda Climate Action and Resiliency Plan* all selected RCP 8.5. The *Kadiköy Municipality Climate Adaptation Action Plan* planned for 1 m of SLR to align with “more radical projections,” while *South Palm Beach* and *Southeast Florida* relied on NOAA's Intermediate‐High scenario. Other assessments, such as the *Vulnerability and Risk Assessment for Gibraltar* and *Climate Change Resilience Strategy* for Nanaimo, used bespoke projections, although details of these methods were often lacking.

This “cherry‐picking” of scenarios mirrors the trend observed by Stults and Larsen (2020) in US adaptation plans and directly conflicts with the IPCC's definition of scenarios [emphasis ours]:

A plausible description of how the future may develop based on a coherent and internally consistent set of assumptions about key driving forces (e.g., rate of technological change, prices) and relationships. Note that scenarios are **neither predictions nor forecasts**, but are used to provide a view of the implications of developments and actions (IPCC 2018).

The IPCC created scenarios as tools to explore Level 4 uncertainties collectively, not as a menu of predictions from which planners can select (Enserink et al. 2013). Yet this predictive treatment of scenarios is widespread. Of the 24 plans that referenced RCPs, 21 (87.5%) described them as “projections,” echoing findings of several case studies in the Netherlands (Enserink et al. 2013).

This misunderstanding may be reinforced by inconsistent IPCC terminology. Working Group I uses “scenarios,” whereas Working Group III uses “modeled emission and mitigation pathways (IPCC 2023b).” Such differences are not trivial, while “scenarios” are typically intended to frame a range of plausible futures without assigning probabilities, the language of “pathways” suggests a more deterministic or forecast‐like trajectory. This subtle shift can inadvertently signal to decision makers that climate projections are more certain than they are, obscuring the exploratory purpose of scenario development. In turn, this risks reinforcing a predict‐and‐act mindset, where plans are built around seemingly definitive futures rather than engaging with the deeper uncertainties that underpin climate change.

Many planners might argue they are still accounting for uncertainty by selecting conservative scenarios as a precautionary approach. Of the selected scenarios, RCP 8.5 is described as the high‐emissions pathway (IPCC 2019), while NOAA's Intermediate‐High is drawn from the upper‐end IPCC AR4 projections (Sweet et al. 2017). The *Kadiköy Municipality Climate Adaptation Action Plan* planned for “more radical projections.” However, this strategy creates an illusion of robustness. While selecting high‐end scenarios may increase robustness to specific hazard levels, it does not provide robustness to uncertainty itself. These conservative scenarios are not true “worst‐case” scenarios and may not be as conservative as decision makers think, as they omit tipping points and cascading failures (Bell et al. 2017). This means that low probability high impact events are not being considered in planning (Kunreuther et al. 2013).

Taking a precautionary approach may also result in overadaptation (Nurmi et al. 2019; Hanemann 2000). Designing for the “worst case” is expensive, and there are significant opportunity costs that may occur (Olutumise et al. 2024). There are also social consequences to overadapting, where large‐scale infrastructure projects and managed retreat can disrupt communities (Khanani et al. 2021; Abu et al. 2024).

This systematic reduction from Level 4 to Level 1 uncertainty for decision making could leave communities vulnerable to climate surprises. Although SLR and associated hazards are widely recognized as deeply uncertain (Sillmann et al. 2020; Stults and Larsen 2020; Bakker et al. 2017; Brock and Xepapadeas 2021; Wong and Keller 2017; Baum 2024; Styczynski et al. 2014; Marchau et al. 2019b; Hulme et al. 2009; Lempert et al. 2024), many decisions (71%) treat them as if they were predictable. Ignoring uncertainty simplifies decision making but degrades the quality and robustness of decisions (Enserink et al. 2013). Given that climate change can be attributed to US$ 143 billion per year in extreme weather events alone and hundreds of thousands of people are being displaced annually (UNDRR 2025; Newman and Noy 2023), this mischaracterization of uncertainty presents a serious risk.

#### 3.3.3 Reduction in Level 4

This mischaracterization of uncertainty, however, is not universal. Two assessments (8.3%, down from 53.8% of general representations) show how deep uncertainty can be integrated into adaptation decision making. These documents employ two concepts from Decision Making under Deep Uncertainty (DMDU): Robustness and Adaptive planning.

Robust plans are designed to work under a multiplicity of futures rather than being optimized for a single future (Fuerth 2009; Quay 2010). *Gibraltar* provides a clear example of using robustness in decision making. Instead of anchoring its strategy to one climate projection, Gibraltar emphasized planning based on vulnerability: “…it is important to base future action on Gibraltar's vulnerability, rather than on the expected impacts from a singular expected future emissions scenario.” This framing seeks to reduce a plan's vulnerabilities regardless of what happens, rather than tailoring it to a single projected future that may not eventuate.

DMDU offers several analytical methods to operationalize this kind of robustness. Robust Decision Making (RDM) uses scenario discovery algorithms to identify the future conditions where a plan may fail, and plans can be updated to better address these conditions (Lempert et al. 2010, 2013). Information Gap Decision Theory uses the “Radii of Stability” to understand the sensitivity of a plan to uncertainty; the plan that loses the least amount of utility for each “increment” of uncertainty is considered most robust (Ben‐Haim 2001, 2019).

Another way to build robustness is with the second concept: Adaptive planning. Adaptive planning accepts that the environment exists in a constant state of flux, requiring plans that change with it (Berke and Lyles 2013; Balducci et al. 2011). This is what *O'ahu* did when they applied an adaptive pathways approach to its climate adaptation. Each action that is suggested in this plan has a timeline (near, medium, long, and far) to allow flexibility as conditions change. This adaptive pathways approach shows how actions can be sequenced and switched between as conditions change.

*O'ahu* used Dynamic Adaptive Policy Pathways (DAPP) (Haasnoot et al. 2019, 2013). However, several other adaptive planning methods exist, including: Dynamic Adaptive Planning (DAP) (Walker et al. 2019) and Multi‐Risk Dynamic Adaptive Policy Pathways (DAPP‐MR) (Schlumberger et al. 2022). Each method is unique, but fundamentally, the adaptive planning process identifies the conditions under which an action may fail and identifies signals and triggers so that a new action can be switched to, ensuring the needs of the community are always met (Lawrence et al. 2020).

DMDU methods are well suited to address the challenges uncertainty brings to climate adaptation (Lempert et al. 2024, 2021; Marchau et al. 2019b); however, only 8.3% of the decisions employed these methods. Two factors may contribute to this gap between acknowledgment and application. First, a lack of understanding of deep uncertainty (46.2% of documents) may prevent practitioners from identifying situations where DMDU tools are most appropriate. Without recognizing that the uncertainties in climate projections, impacts, and socioeconomic pathways cannot be reliably quantified, decision makers may continue to assume that a single “best estimate” is sufficient.

Second, even among those aware of deep uncertainty, familiarity with the practical tools and methods available remains low (Lawrence et al. 2019; Bonjean Stanton and Roelich 2021; Woodruff 2016). Techniques such as RDM, DAPP, and Information Gap Decision Theory require specialized knowledge, data processing capabilities, and in some cases, cultural shifts in planning practice (Bonjean Stanton and Roelich 2021). In the absence of this awareness or capacity, decision makers often default to established, projection‐based methods.

### 3.4 Methodological Limitations

The results demonstrate a clear and systematic misrepresentation of uncertainty in climate risk and vulnerability assessments globally. Several features of the document selection process may limit the generalizability of these findings.

Documents were sourced from the CDP database, which skews toward cities that are already actively engaged in climate reporting. This suggests that the 54% of documents correctly representing deep uncertainty is likely an optimistic estimate; a sample drawn from less climate‐engaged municipalities would probably reveal an even wider gap between scientific understanding and planning practice.

Restricting the sample to publicly available documents may skew results in the other direction. Cities conducting more technically sophisticated work may not publish it as it may not be accessible for the general population. Instead, cities will often publish plain language summaries that may obscure how uncertainty is incorporated into their internal processes.

Finally, while SLR provides a well‐suited case study given its deep uncertainty and the maturity of its scenario frameworks, it is also the main focus of climate reporting where the presence of uncertainty is well‐established. Uncertainty representation for hazards such as extreme heat may differ considerably. Future research could explore whether similar patterns emerge in other deeply uncertain climate hazards, such as extreme precipitation, wildfires, or heatwaves. Taken together, these limitations suggest that the disconnect between scientific understanding and planning practice identified here is likely a conservative estimate of a more pervasive problem.

## 4 Practical Implications

Our analysis reveals a concerning disconnect between scientific understanding of climate uncertainty and planning practice. While 54% of climate risk and vulnerability assessments acknowledge SLR as deeply uncertain, only 8.3% of decisions actually incorporate this deep uncertainty into their decision‐making processes. Instead, 71% of adaptation decisions rely on single projections, thereby treating deeply uncertain phenomena as if they were predictable. This gap between recognition and application points to two critical areas requiring intervention: improving the communication of uncertainty and applying DMDU methods to account for this uncertainty.

### 4.1 Improving Communication of Uncertainty

The finding that nearly half (46%) of climate risk and vulnerability assessments fail to recognize SLR as deeply uncertain indicates a fundamental communication problem. This disconnect between climate science and planning practice stems largely from a disconnection from scenario planning, the terminology around the scenarios, and inadequate characterization of uncertainty. Each of these contributes to a tendency for decision makers to misinterpret scenarios as predictions rather than exploratory tools, thereby reinforcing a predict‐and‐act mindset.

#### 4.1.1 Disconnection From Scenario Planning

There is misalignment between the IPCC scenarios and the wider scenario planning literature. The SRES deliberately presented four scenarios with no central case (Nakicenovic and Swart 2000), whereas AR6 included five SSPs. This goes against the recommendation of many scenario planning scholars to avoid central scenarios (Goodspeed 2020; Meristö 2017). In doing so, the IPCC has opened the door for decision makers to misinterpret the scenarios using the “median” scenario to mean the most likely. This could be addressed by including an additional scenario or reverting to four scenarios in line with the scenario planning literature (Goodspeed 2020; Meristö 2017).

There also needs to be educational resources that come with the climate scenarios to convey that the scenarios are a planning tool rather than a set of projections that can be selected from. Currently, decision makers are misusing the scenarios by selecting a single scenario and designing for that (71%). This could be addressed by being specific about the function of scenarios and pointing decision makers toward exploratory scenario planning resources such as: Stapleton (2020) and Goodspeed (2020).

#### 4.1.2 Scenario Terminology

Part of the reason that climate scenarios are not being treated as scenarios is the terminology used by the IPCC. The use of both “scenarios” and “modeled emission and mitigation pathways” (IPCC 2023b) within IPCC reports creates ambiguity. This terminology shift implies a degree of certainty as the outcomes have been modeled rather than plausible storylines designed to explore uncertainty. Consistent use of the word “scenario” instead of pathways would help reinforce that these are not projections (as 87.5% of documents did) but rather a tool to explore uncertainty.

#### 4.1.3 Characterizing Uncertainty

Attempts to standardize uncertainty communication have been made that provide language on how to communicate the likelihood and the confidence of information (Mastrandrea et al. 2010). However, this limits how uncertainty can be represented. Instead, there needs to be a shift beyond quantifying uncertainty to characterizing it, in line with Walker et al. (2003), who distinguish uncertainty by its nature, location, and level. Such an approach would provide a more nuanced account of the uncertainties that underpin climate change and make explicit where knowledge is limited and outcomes are unpredictable. A stronger emphasis on characterising uncertainty would enable policies that are both more transparent about scientific limits and identify when DMDU methods may be required.

### 4.2 DMDU

While just over half of the documents recognized that SLR was deeply uncertain, only 8.3% (n=2) of decisions adequately accounted for deep uncertainty by using DMDU concepts. Most plans acknowledge SLR as a challenge, yet continue to rely on deterministic assumptions or single‐scenario designs that leave them vulnerable if futures unfold differently than expected. By contrast, the two cases that did incorporate DMDU demonstrate that it is both feasible and valuable to embed deep uncertainty concepts directly into decision‐making processes.

The *Vulnerability and Risk Assessment* for Gibraltar used the concept of robustness to address uncertainty. Robustness is used to design plans to perform adequately regardless of the future that eventuates. This is a key concept in DMDU methods such as RDM (Lempert et al. 2013), Engineering Options Analysis (EOA) (Smet 2017), and Scenario Planning (Avin and Goodspeed 2020). These methodologies stress testing plans across the range of plausible futures, identifying weaknesses, and designing strategies that perform well regardless of how conditions evolve.

Another pathway to robustness is adaptive planning. Adaptive strategies are designed to change in response to evolving conditions or new information. The *Climate Ready O'ahu* adaptation plan demonstrated this through the use of DAPP. DAPP structures plans as metro‐maps showing the range of some uncertain condition actions are effective over and what other actions can be switched to in the case that an action is about to fail (Haasnoot et al. 2019). Other frameworks, such as DAPP‐MR (Schlumberger et al. 2022) and DAP (Walker et al. 2019) offer similar approaches.

The IPCC has begun incorporating these concepts into its assessments (Lempert et al. 2024), a trend that, if sustained, can strengthen awareness of the relationship between the deep uncertainties inherent in climate change and the decision‐support tools designed to address them. In this context, *Decision Making under Deep Uncertainty: From Theory to Practice* (Marchau et al. 2019a) provides a comprehensive overview of the key concepts and methods underpinning DMDU approaches, offering a valuable reference for both researchers and practitioners.

## 5 Conclusions

This study reveals a critical disconnect between scientific understanding of climate uncertainty and its application in adaptation planning practice. Through a structured review of 39 climate risk and vulnerability assessments from around the world, using SLR as a case study, we demonstrate that the deep uncertainty inherent in climate change is systematically misrepresented in planning documents. Our analysis shows that only 54% of climate risk and vulnerability assessments correctly represent SLR as deeply uncertain (Level 4a or 4b uncertainty). This finding is concerning, given the widespread scientific consensus that SLR is deeply uncertain.

Perhaps more concerning is the pattern that emerges when uncertainty representations are translated into actual decisions. While 54% of documents acknowledge deep uncertainty in their analysis, only 8.3% of policy recommendations and planning decisions incorporate this understanding. Instead, 71% of adaptation decisions rely on single projections, effectively treating a deeply uncertain phenomenon as predictable. This represents a fundamental misapplication of climate scenarios, which were designed to be used collectively as a tool for exploring uncertainty and understanding the vulnerabilities of a plan, not as a menu of projections from which planners can select.

This tendency to reduce deep uncertainty (Level 4) to deterministic planning (Level 1) has serious consequences for community resilience and resource allocation. This misrepresentation opens the door for predict‐and‐act methods, which undermine the effectiveness of climate adaptation by narrowing the range of futures considered. Traditionally, one way to account for this uncertainty was through the precautionary principle. However, the precautionary principle by selecting the highest scenario only creates an impression of robustness. These scenarios overlook low‐probability, high‐impact events or drive overadaptation, wasting resources, and disrupting communities unnecessarily. Ensuring deep uncertainty is recognized through the entire decision‐making process requires intervention at two levels: first, improving recognition of deep uncertainty, and second, strengthening the capacity to apply DMDU methods.

In order to improve the recognition of deep uncertainty, climate science communities must move beyond quantifying uncertainty to characterizing it. Characterization could take the form of established frameworks like the Levels of Uncertainty. The terminological inconsistencies in IPCC reports must also be addressed. Presenting scenarios as “modeled emission pathways” shifts the framing from plausible futures to a calculated projection. This should be coupled with clearer guidance on how to use scenarios as exploratory tools.

There is also a need to support decision makers build the capacity for DMDU methods. The two cases in our sample that successfully incorporated deep uncertainty—Gibraltar's vulnerability‐based approach and O'ahu's adaptive pathways strategy–demonstrate that robust DMDU is achievable. However, the scarcity of such applications (8.3%) indicates a need for greater awareness and training in DMDU methods. Continued research into supporting the uptake of DMDU approaches in climate planning should focus on identifying practical methods for integrating deep uncertainty into decision making while ensuring accessibility for policymakers who may prefer clearer, more deterministic guidance.

Our findings demonstrate a systematic gap between the way climate science understands uncertainty and how it is incorporated into planning practice. This gap has profound implications as adaptation decisions made today will shape community resilience for decades, yet many are being guided by assessments that present SLR with a false sense of precision. With billions of dollars in infrastructure at stake, the risks of underpreparation or overadaptation are too significant to ignore. Addressing this gap must therefore be a priority for both scientific and planning communities.

The tools to close this gap already exist. DMDU methods have been successfully applied in select cases, demonstrating that robust and adaptive strategies are achievable. The broader challenge is integrating these approaches into routine planning practice and supporting decision makers to characterize rather than oversimplify uncertainty by simply attempting to quantify it. By acknowledging and working with deep uncertainty, adaptation planning can move beyond simplistic predictive models and precautionary assumptions toward strategies that are genuinely resilient in the face of climate change.

## Funding

TL, PC: MBIE Smart Idea: Innovating climate risk assessment: A system‐wide, geospatial approach for councils and communities. TL: Rutherford Discovery Fellowship: Incorporating cascading risk and multiple uncertainties into climate adaptation planning. KH: United States‐New Zealand Fulbright Research Award

## Conflicts of Interest

TL has interested in the risk company Urban Intelligence and its software Resilience Explorer.

## Appendix A List of Documents Reviewed

**Table:**

City	Title	Year
Alameda, CA, USA	Alameda Climate Action and Resiliency Plan	2019
Auckland, New Zealand	Climate Change Risks in Auckland	2019
Barcelona, Spain	Climate Emergency Action Plan for 2030	2021
Bloomington, IN, USA	Climate Risk and Vulnerability Assessment	2020
Boca Raton, FL, USA	Multi‐jurisdictional Climate Change Vulnerability Assessment	2021
Boynton Beach, FL, USA	Multi‐jurisdictional Climate Change Vulnerability Assessment	2021
Bristol, England	Bristol One City Climate Strategy: Preliminary Climate Resilience Assessment	2020
Can Tho, Vietnam	Climate change vulnerability assessment for Can Tho city by a set of indicators	2019
Cape Town, South Africa	Inception Report	2018
Cape Town, South Africa	Vulnerability and Hazard Assessment Report	2019
Dunedin, New Zealand	Otago Climate Change Risk Assessment	2021
Durban, South Africa	Turning future climate risk into resilience: Climate Projections and Risk	2019
	Assessment for eThekwini Municipality	
Freetown, Sierra Leone	Sierra Leone Multi‐City Hazard Review and Risk Assessment	2018
Gibraltar	Vulnerability and Risk Assessment for Gibraltar	2022
Glasgow, Scotland	Glasgow Climate Adaptation plan	2022
Hobart, Australia	Sustainable Hobart Action Plan	2020
Honolulu, Hi, USA	Climate Ready O'ahu Risk Assessment Results	2020
Houston, Tx, USA	Climate Impact Assessment for the City of Houston	2020
Istanbul, Turkey	Istanbul Climate Change Action Plan	2021
Kadiköy, Turkey	Kadiköy Municipality Climate Adaptation Action Plan Report 2	2018
Kisumu County, Kenya	Kisumu Country climate change vulnerability assessment report	2020
Lagos, Nigeria	Lagos State Climate Risk Assessment	2021
London, England	Climate change risks for London	2019
Los Angeles, CA, USA	City of Los Angeles 2018 Local Hazard Mitigation Plan	2018
Manhattan Beach, CA, USA	Sea Level Rise Risk, Hazards, and Vulnerability Assessment, City of	2021
	Manhattan Beach	
Medford, OR, USA	Medford Climate Change Vulnerability Assessment	2019
Miami, FL, USA	Unified Sea Level Rise Projection Southeast Florida	2019
Nanaimo, Canada	Climate Change Resilience Strategy	2020
New Plymouth, New Zealand	Climate change projections and impacts for Taranaki	2022
Quezon, Philippines	Climate and Disaster Risk Assessment Report for Quezon City	2022
Ramallah, Palestine	Palestine Municipality of Ramallah: Sustainable Energy Access& climate action plan	2022
Reading, England	Reading Climate Change Adaptation Plan	2019
Saanich, Canada	Climate Preparedness and Adaptation Strategy	2021
Saanich, Canada	Preliminary Strategic Climate Risk Assessment for British Columbia	2019
San Francisco, CA, USA	City and County of San Francisco Hazards and Climate Resilience Plan March 26, 2020	2020
Singapore	Charting Singapore's Low‐Carbon and Climate Resilient Future	2020
Superior, CO, USA	2022–2027 Boulder Hazard Mitigation Plan	2022
Unley, Australia	Climate Change Adaptation Governance Assessment: Report for the City of Unley	2021
Vancouver, Canada	Climate Change Adaptation Strategy	2018
Wellington, New Zealand	Coastal hazards and sea‐level rise in Wellington City	2021

## Appendix B Edge Cases From the Review

The first edge case was found in the report for Lagos, Nigera where it stated *“Badagry and Epe local Government areas will be inundated by the year 2100.”* When taken at face value, this statement suggests that there is a large range of possibilities of when these areas will be inundated (2021–2100) and therefore should be classified as a level 4a uncertainty. However, we did not want to conflate the lack of precision in language and the lack of precision due to the uncertainty in SLR. The source specifically cited for these statistics could not be found, but it appears the analysis was conducted using scenarios and as such it was classified as level 3 Epsilon Innovation Group (2017).

The second edge case was for Manhattan Beach. In this report, they analyzed, *“…four storm scenarios were analyzed with CoSMoS under ten sea level rise scenarios, including existing sea level.”* They suggest they had 10 SLR scenarios, indicating a level 3 uncertainty. However, what was called scenarios was increments of SLR from 0 to 5 m every 0.25 m with no timings attached. Increments without timings made this a 4a uncertainty.

The inception report for Cape Town presented another edge case. *“Sea level rise and coastal erosion [were] assessed according to 3 scenarios”* again implying a level 3 uncertainty. The first scenario is the present‐day worst‐case scenario, the second is the end of the next decade scenario, and the third is a polar ice sheet collapse scenario. The first two scenarios are point estimates in time and therefore act more as a level 1 uncertainty. The ice sheet collapse scenario provided another narrative exploring the uncertainties in ice sheet dynamics without prescribing a time for this. Between the reviewers, it was agreed that this instance would be classified as both level 1 and level 3.

The Climate Change Risks for London document gave us our final edge case. Possible SLR was again presented using two different levels of uncertainty; *“if carbon emissions are kept low is very likely to be in the range 0.29 m to 0.70 m. If emissions remain at current levels, the range is very likely to be 0.53 m to 1.15 m.”* The low emissions and current emissions scenarios were classified as a level 3 uncertainty, and the likely ranges of SLR around these scenarios were classified as a level 2 uncertainty. This is how the IPCC presents the uncertainty around their RCP/SSPs. It is possible that when others have presented a range of possible SLR they have misrepresented the “likely” or “very likely” resulting in a level 4a classification on what should have been a level 2 classification.
